# Multicenter Validation of a Machine Learning Model for Surgical Transfusion Risk at 45 US Hospitals

**DOI:** 10.1001/jamanetworkopen.2025.17760

**Published:** 2025-06-27

**Authors:** Sunny S. Lou, Sayantan Kumar, Charles W. Goss, Michael S. Avidan, Sachin Kheterpal, Thomas Kannampallil

**Affiliations:** 1 Department of Anesthesiology, Washington University School of Medicine, St Louis, Missouri; 2 Institute for Informatics, Data Science, and Biostatistics, Washington University School of Medicine, St Louis, Missouri; 3 Department of Computer Science and Engineering, Washington University School of Medicine, St Louis, Missouri; 4 Department of Anesthesiology, University of Michigan, Ann Arbor

## Abstract

**Importance:**

Accurate estimation of surgical transfusion risk is important for perioperative planning and effective resource allocation. Most machine learning models in health care are not validated or perform poorly in external settings.

**Objective:**

To externally validate a publicly available machine learning algorithm (Surgical Personalized Anticipation of Transfusion Hazard [S-PATH]) to estimate red cell transfusion during surgery within a national sample of hospitals.

**Design, Setting, and Participants:**

This retrospective cohort study evaluated all surgical cases performed in 2020 or 2021 at 45 US hospitals participating in the Multicenter Perioperative Outcomes Group. Obstetric and nonoperative cases were excluded. Data analysis was performed from February 2023 to March 2025.

**Exposures:**

At each hospital, S-PATH was used to estimate surgical transfusion risk using patient- and procedure-specific characteristics without local retraining. A baseline model representing the standard-of-care maximum surgical blood ordering schedule (MSBOS) approach, which omits patient factors, was used for comparison. Risk thresholds above which a type and screen would be recommended were set for 96% sensitivity. Performance was evaluated at each hospital separately.

**Main Outcomes and Measures:**

The primary outcome was the difference in the percentage of patients with type and screen order recommendations between S-PATH and MSBOS at each hospital. The secondary outcome was area under the receiver operating characteristic curve (AUROC).

**Results:**

In this cohort study of 3 275 956 surgical cases (median [IQR] age, 57 [40-69] years; 53.1% female) performed at 45 hospitals (28 of 45 academic [62.2%]), S-PATH recommended type and screen orders for a median (IQR) of 32.5% (25.8%-42.0%) of cases, whereas the MSBOS approach recommended type and screens for a median (IQR) of 51.6% (46.9%-61.1%) of cases for the same sensitivity (median [IQR] difference, 17.9 [14.8-24.9] absolute percentage points). The median (IQR) S-PATH AUROC was 0.929 (0.915-0.946), whereas the median (IQR) MSBOS AUROC was 0.857 (0.822-0.884).

**Conclusions and Relevance:**

In this cohort study of 45 hospitals, a personalized surgical transfusion risk prediction algorithm demonstrated external validity and discrimination. S-PATH was consistently more effective than standard care, suggesting its potential for use as a perioperative clinical decision support tool.

## Key Points

**Question:**

Does a publicly available personalized machine learning model for surgical transfusion risk consistently outperform the standard-of-care approach for guiding preoperative type and screen orders across diverse health systems?

**Findings:**

In this cohort study of 45 US hospitals, a personalized model recommended type and screen orders for a median 17.9 absolute percentage point fewer patients than the standard-of-care approach despite equivalent 96% sensitivity.

**Meaning:**

The personalized algorithm demonstrated robust external validity across a diverse cohort of hospitals, suggesting its potential to improve resource allocation if broadly implemented as a perioperative clinical decision support tool.

## Teaser

This cohort study evaluates the generalizability and potential clinical application of a publicly available machine learning algorithm to estimate red cell transfusion during surgery within a national sample of hospitals.

## Introduction

Blood transfusion is a lifesaving therapy in the perioperative setting. It is estimated that approximately 20% of the red cells transfused in the US are administered during surgery.^1^ Safe administration of intraoperative blood products requires considerable planning and multiple steps that can take hours to days, including blood typing and antibody screening (type and screen), confirmatory testing, crossmatching to identify compatible units, and delivery to the operating room.^2^ In contrast, delays on the order of minutes for blood availability in the operating room can be life-threatening. Therefore, adequate preoperative preparation for intraoperative blood transfusion needs is critically important for patient safety during surgery. However, as highlighted by the Choosing Wisely campaign,^3^ unnecessary preparation for patients at low risk of transfusion is wasteful and increases the cost of care.^4^ Unnecessary crossmatching has also been associated with an increased risk for discarded blood products,^5^ a substantial problem given the threatened sustainability of the nation’s blood supply.^6,7^ There is an acute public health need for tools that accurately estimate the risk of surgical transfusion to guide clinical decision-making.

The current standard of care is to estimate transfusion risk and determine preoperative blood orders based exclusively on the planned procedure, typically using a maximum surgical blood ordering schedule (MSBOS), a nomogram that lists recommended orders for each procedure based on historical hospital-specific transfusion rates for each procedure.^8^ Surgical transfusion risk substantially depends on patient-specific characteristics, such as preoperative anemia and blood volume, which are not accounted for in the MSBOS.^9,10^ We previously developed, published, and made publicly available a personalized surgical transfusion risk prediction artificial intelligence (AI) model (Surgical Personalized Anticipation of Transfusion Hazard [S-PATH]) that accounts for both procedure- and patient-specific characteristics and tailored it to guide decision-making for presurgical blood orders.^11^ S-PATH is a gradient boosting machine model trained based on 3 million surgical cases in the National Surgical Quality Improvement Program (NSQIP) database and validated at a single academic medical center, where it showed potential to reduce both missed and unnecessary presurgical blood orders.^11^ Further validation of model discrimination was also performed using a limited subset of surgical cases submitted to the NSQIP by participating hospitals.^12^

The primary objective for this study was to evaluate the generalizability and potential clinical application of S-PATH as a clinical decision support tool across a broader set of procedures performed at a diverse cohort of academic and community hospitals within the US. We simulated S-PATH prospective implementation at each hospital and assessed its predictions for presurgical blood orders compared with the standard-of-care MSBOS approach. Although many machine learning models require resource-intensive retraining or fine-tuning at each hospital to achieve adequate performance, we hypothesized that S-PATH would perform well even without such efforts.

## Methods

This retrospective cohort study was approved by the institutional review board of Washington University with a waiver of informed consent because the research was considered minimal risk. A study protocol for cohort selection, data processing, and statistical analysis was registered on Open Science Framework before analysis.^13^ This study is reported following the Transparent Reporting of a Multivariable Prediction Model for Individual Prognosis or Diagnosis (TRIPOD) AI and Strengthening the Reporting of Observational Studies in Epidemiology (STROBE) reporting guidelines.^14,15^

### Data Sources

This study used data extracted from the Multicenter Perioperative Outcomes Group (MPOG) Standardized Data File.^16^ MPOG is a consortium of more than 60 academic and community medical centers across the US that submit perioperative electronic health record data monthly to a central data registry. All data are manually mapped to standardized concepts, and data validation checks are performed before submission; a subset of data is manually audited quarterly to ensure quality.^17^ The Standardized Data File contains information on the highest-quality variables in the MPOG registry after additional data cleaning steps have been performed to remove data artifacts and harmonize reporting units. Information on observed type and screen orders was not reliably available as part of this dataset. Information on patient race was self-reported by each medical center.

This study used data from the MPOG Standardized Data File, version 2021, which contained information on all surgical cases performed at MPOG-participating centers from January 1, 2016, through December 31, 2021. Because this study aimed to simulate prospective implementation of S-PATH starting in 2020, the data were split into a historical cohort containing data from 2016 through 2019 (strictly used for computing historical hospital-specific procedure-specific transfusion rates as described below) and a validation cohort containing data from 2020 and 2021 (used for evaluating S-PATH performance).

### Cohort Definition

All hospitals that had data available for both the historical and validation cohorts were included. Four hospitals were excluded due to data quality issues.^13^ For each hospital, case-level inclusion criteria were chosen to be broad to best imitate implementation of S-PATH as clinical decision support for all surgical cases. Specifically, all cases were included unless they were obstetric, nonoperative, or related to organ donation (ie, American Society of Anesthesiology [ASA] physical status score 6) because different transfusion considerations apply for these specialized populations. A full participant flow diagram illustrating all inclusion and exclusion criteria is shown in eFigure 1 in Supplement 1.

### Data Preprocessing

S-PATH relies on the following variables to compute predicted risk of red cell transfusion during surgery^11^: patient age, weight, height, sex, presence of specific comorbidities (hypertension, congestive heart failure, smoking, chronic obstructive lung disease, dialysis, and diabetes, defined using Elixhauser comorbidities), preoperative laboratory values (hematocrit, platelet count, international normalized ratio, partial thromboplastin time, and creatinine, sodium, albumin, and bilirubin concentrations), whether the procedure was elective, and the historical transfusion rate for the procedure. These historical procedure-specific transfusion rates were used to adapt model predictions for local context without requiring model retraining. Specifically, historical procedure-specific transfusion rates were computed separately for each hospital using data from that hospital’s historical cohort. Procedures were grouped by their predicted anesthesia *Current Procedural Terminology* (*CPT*) code based on a previously published algorithm that matches *CPT* codes to procedural free text.^18^ For each hospital and *CPT* code, the rate of red cell transfusion in the historical cohort was calculated (eg, for *CPT* code 01402 at hospital A, 5 of 100 cases [5%] in the historical cohort received red cell transfusion). This hospital-specific, procedure-specific transfusion rate was used as an input variable for the model for all cases at that hospital with that *CPT* code in the validation cohort. We had previously shown that at least 50 historical cases were necessary to establish a reliable historical procedure-specific transfusion rate.^12^ Therefore, cases in the validation cohort with an historical hospital-specific, procedure-specific transfusion rate relying on fewer than 50 historical examples were excluded (69 363 of 3 345 319 total cases [2.1%]). Of note, these historical hospital-specific, procedure-specific transfusion rates are also required during the creation of a conventional MSBOS.^8^ As described in our previous work,^11^ these input variables were selected for their widespread availability in the preoperative period, where we envision S-PATH might be used in routine clinical care.

### Model Evaluation

S-PATH performance was evaluated within the validation cohort at each hospital separately; the entire S-PATH pipeline was applied without modification, including data normalization and missing value imputation, using code available from a public repository.^19^ No retraining or fine-tuning was performed.

A baseline model representing the conventional MSBOS approach was also evaluated at each hospital to serve as a comparator to the current standard of care. The MSBOS is essentially a chart that lists the recommended presurgical blood orders for different procedures.^20,21^ Current transfusion guidelines support the data-driven creation of a MSBOS based on the historical transfusion rates for each procedure, such as recommending a type and screen for procedures with an historical transfusion risk greater than 5%.^22,23^ The baseline model uses the historical hospital-specific, procedure-specific transfusion rates as the predicted risk, analogous to how this information is used to develop MSBOS recommendations.^8^

Because the intention was to evaluate S-PATH’s potential as a clinical decision support tool for presurgical type and screen decisions, decision thresholds (ie, transfusion risk above which the model recommends a presurgical type and screen) for both models were set to achieve 96% sensitivity for detecting patients needing transfusion during surgery. This threshold was chosen based on expert opinion to balance the asymmetric harms of false-negative predictions (patients needing transfusion without a type and screen) and false-positive predictions (patients with type and screens who do not need transfusion) and is largely in line with surveyed clinician opinions.^24^

### Outcomes

All outcomes were assessed at the level of the individual hospital. The primary outcome was the difference in the frequency of type and screen recommendations between S-PATH and the MSBOS approach. This outcome was chosen to highlight the clinically meaningful difference between the 2 approaches given that decision thresholds were set for equivalent sensitivity. Secondary outcomes included area under the receiver operating characteristic curve (AUROC) as a measure of overall model discrimination and calibration plots as a measure of overall calibration. Model evaluation was performed using Python, version 3.9.13 (Python Software Foundation).

### Statistical Analysis

Hospital characteristics and model performance within each hospital were summarized using descriptive statistics (ie, median [IQR] for continuous variables and number [percentage] for categorical variables). Correlations between individual hospital-level characteristics and S-PATH performance were explored using Pearson correlation coefficients. Hospital-level characteristics available included annual surgical volume, percentage of patients with an ASA physical status classification or 3 or higher (reflecting patient complexity), percentage of procedures with an ASA base unit value of 7 or higher (ie, physiologically complex or larger cases with higher risk for surgical bleeding),^25^ percentage of patients receiving red cell transfusion, and degree of adherence to evidence-based transfusion practices (measured using the MPOG quality measure TRAN01,^26,27^ which captures the frequency of measuring the hemoglobin or hematocrit within the 90 minutes before transfusion). Statistical analysis was performed using R software, version 4.1.2 (R Foundation for Statistical Computing. A 2-sided *P* < .05 was considered to be statistically significant. Data analysis was performed from February 2023 through March 2025.

## Results

### Cohort Characteristics

A total of 45 hospitals and 3 275 956 surgical cases (median [IQR] age, 57 [40-69] years; 53.1% female and 46.9% male) were included in this study (Table 1; eTables 1-2 in Supplement 1). Across the included hospitals, median (IQR) surgical volume was 31 519 (14 477-53 948) cases per year, with a median (IQR) of 1.5% (0.6%-2.8%) of cases requiring red cell transfusion. A total of 28 of 45 hospitals (62.2%) were affiliated with a medical school, and 25 of 45 hospitals (55.6%) had more than 500 beds. Procedural and patient complexity varied across the included hospitals, with a median (IQR) of 51.2 (45.8-57.6) patients having an ASA physical status score of 3 or higher and a median (IQR) of 11.6 (9.1-15.2) cases having an ASA base unit value of 7 or higher (ie, considered a physiologically complex surgery).

**Table 1.** Demographic Characteristics of the 45 Study Hospitals

Characteristic	Finding
Annual surgical volume, median (IQR), No.	31 519 (14 477-53 948)
Cases requiring transfusion, median (IQR), %	1.5 (0.6-2.8)
Medical school affiliation, No. (%)	28 (62.2)
Hospital bed size, No. (%)	
100-199	4 (8.9)
200-299	2 (4.4)
300-399	5 (11.1)
400-499	9 (20.0)
≥500	25 (55.6)
Cases with ASA physical status classification ≥3, median (IQR), %^a^	51.2 (45.8-57.6)
Cases with ASA base units ≥7, median (IQR), %^b^	11.6 (9.1-15.2)
Cases with procedure-specific risk >1%, median (IQR), %	28.0 (14.4-38.3)
Adherence to national transfusion quality metric, median (IQR), %^c^	56.8 (46.9-62.9)

Abbreviation: ASA, American Society of Anesthesiology.

^a^ ASA physical status classifications are assigned based on the patient (range, 1-5) and represent patient complexity.

^b^ ASA base units are assigned based on the procedure (range, 1-30) and represent procedural complexity.

^c^ Percentage of patients receiving transfusion with a hemoglobin check within 90 minutes before transfusion.

### Model Performance

Across the 45 hospitals, the median (IQR) AUROC for S-PATH was 0.929 (0.915-0.946), whereas the median (IQR) AUROC for the baseline MSBOS approach was 0.857 (0.822-0.884) (Figure, A and Table 2). The S-PATH AUROC was greater than 0.91 for more than 75% of the hospitals examined. Calibration plots comparing predicted with observed transfusion risk are shown in eFigure 2 in Supplement 1.

**Figure.**
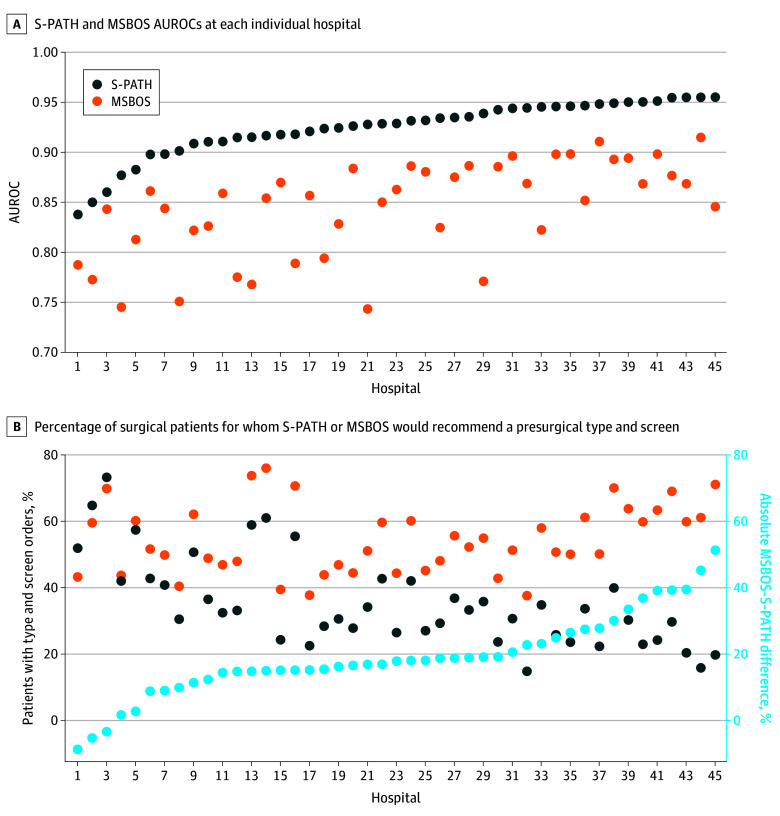
Surgical Personalized Anticipation of Transfusion Hazard (S-PATH) Performance Compared With the Conventional Maximum Surgical Blood Ordering Schedule (MSBOS) Approach at Each Hospital A, S-PATH (navy blue dot) and MSBOS (orange dot) area under the receiver operating characteristic curve (AUROC) at each individual hospital. Hospitals are arranged on the x-axis in ascending order by S-PATH AUROC. B, Percentage of surgical patients for whom S-PATH (navy blue dot) or MSBOS (orange dot) would recommend a presurgical type and screen at each hospital. The absolute differences in percentage of patients with type and screen recommendations between the S-PATH and MSBOS approaches at each hospital are shown as light blue dots (y-axis shown at right). Hospitals are arranged on the x-axis in ascending order by absolute percentage difference in type and screen orders.

**Table 2.** S-PATH Performance Compared With the Standard-of-Care MSBOS Approach^a^

Metric	Median (IQR) (N = 45)
AUROC	
S-PATH	0.929 (0.915-0.946)
MSBOS	0.857 (0.822-0.884)
Sensitivity	
S-PATH	0.959 (0.958-0.960)
MSBOS	0.956 (0.939-0.960)
Cases with type and screen orders, %	
S-PATH	32.5 (25.8-42.0)
MSBOS	51.6 (46.9-61.1)
Absolute difference in type and screen orders, %	17.9 (14.8-24.9)

Abbreviations: AUROC, area under the receiver operating characteristic curve; MSBOS, maximum surgical blood ordering schedule; S-PATH, Surgical Personalized Anticipation of Transfusion Hazard.

^a^ S-PATH and MSBOS performance was evaluated at each hospital separately. Summary statistics of performance (median [IQR]) are aggregated over the 45 included hospitals.

To achieve 96% sensitivity for detecting patients needing transfusion during surgery, S-PATH recommended type and screens for a median (IQR) of 32.5% (25.8%-42.0%) of patients across the 45 hospitals; in contrast, the baseline MSBOS approach recommended type and screen orders for a median (IQR) of 51.6% (46.9%-61.1%) of patients to achieve a similar sensitivity (median [IQR] difference, 17.9 [14.8-24.9] absolute percentage points) to S-PATH (Figure, B and Table 2). This difference translated to 631 655 fewer type and screens across the entire study cohort or a cost savings of approximately $10 million assuming $15.75 reimbursed for each type and screen as stipulated by the 2025 Medicare Clinical Laboratory Fee Schedule^28^; however, the true activity-based cost for a type and screen has been estimated to be several-fold higher.^29,30,31^ S-PATH performance did not meaningfully differ when stratified by race (eFigure 3 and eTable 3 in Supplement 1).

### Hospital-Level Characteristics and Model Performance

To assess whether there were patterns in the hospitals for which S-PATH performed poorly, correlations between hospital-level characteristics and S-PATH performance were explored (eFigure 4 and eTable 4 in Supplement 1). No hospital-level characteristics were significantly correlated with the difference in frequency of type and screens between S-PATH and the MSBOS approach (primary outcome). However, hospitals with higher annual surgical volume tended to have higher S-PATH AUROC (Pearson *r* = 0.456; 95% CI, 0.187-0.661; *P* = .002). In general, S-PATH performance appeared more variable at smaller hospitals with lower surgical volume.

## Discussion

We evaluated the performance of S-PATH, a personalized surgical transfusion risk prediction model, within a diverse cohort of 45 academic and community US hospitals serving more than 3 million surgical patients. S-PATH consistently outperformed the standard-of-care MSBOS approach at most hospitals, with higher overall discrimination and requiring fewer type and screen orders to adequately detect 96% of patients who subsequently required transfusion. These findings suggest S-PATH’s generalizability and robustness and provide evidence to support its potential for pragmatic clinical value in improving resource allocation if implemented broadly.

### Strengths 

This study has several strengths. Generalizability has been a long-sought-after and controversial subject in the field of AI for health care literature. Many machine learning models have failed to generalize in external validation for a number of reasons,^32,33,34,35^ including model overfitting and dataset shift between the training and test data, usually resulting from differences in patient population or clinical practice patterns across location and time.^36,37^ The inability of machine learning models to generalize has limited the uptake of AI in health care because the health care system resource commitment and expertise needed to train or fine-tune home-grown models can be substantially greater than that needed to implement an existing model.

We showed that S-PATH demonstrated relatively consistent performance across a diverse range of hospitals without requiring model retraining at each institution. These results suggest that S-PATH may be useful immediately for many health care systems, which could potentially reduce the cost of implementation. There are several potential contributors to S-PATH’s robustness. First, we used the historical event rate (ie, transfusion rate) for each procedure as an input variable to the model, which served as a form of transfer learning to allow model customization to specific hospitals; this approach also enables future updates to adjust for changes in transfusion practices over time. Second, it was trained on a large, multi-institutional, high-quality dataset consisting of manually validated data elements, which perhaps increased the likelihood of the model learning robust relationships between the input variables and the transfusion outcome.^11^ Indeed, the variables that most contribute to the model’s decision-making (patient hematocrit, procedure-specific transfusion rate, and laboratory indicators of coagulopathy) are consistent with clinician intuition.^11^ Third, S-PATH is a relatively simple model, with only 20 input variables carefully chosen for clinical relevance, practicality, and ease of extraction from electronic health record data. By limiting the feature space, we likely reduced the risk of overfitting (ie, the risk of identifying spurious relationships among the variables). Use of fewer predictor variables also facilitates practical implementation because fewer variables need to be mapped and cleaned for the model pipeline within the electronic health record. It is possible that S-PATH performance could be further improved by retraining the model within individual hospitals; however, such retraining may require additional computational expertise and resources that may not be available to many health systems. In this study, we demonstrated that S-PATH can achieve effective results even without hospital-specific retraining, potentially lowering the cost for model implementation.

Other strengths of this study include its scale and use of clinically meaningful evaluation metrics. External validation of machine learning models has been largely limited by data availability, and most studies^38,39,40,41,42,43^ have focused on validation within a handful of institutions. In our study, we leveraged a large, multi-institutional, perioperative data registry to evaluate S-PATH performance across 45 US hospitals and more than 3 million patients. Data-sharing consortiums are critical for large-scale model development and validation, which are necessary to support widespread uptake of AI in health care. In addition, we chose evaluation metrics focused on illustrating the direct potential clinical outcomes of S-PATH implementation. Specifically, our primary outcome focused on measuring the frequency of type and screen orders at a specific high level of sensitivity, which acknowledged the asymmetric harms of false-positive and false-negative results for this prediction problem. In addition, we compared S-PATH with the current standard-of-care MSBOS approach, which allowed us to demonstrate the pragmatic potential for S-PATH to improve resource allocation and reduce health care costs through a median 17.9% absolute reduction in the percentage of patients with type and screen orders. As a publicly available algorithm with syntax available for inspection and modification, S-PATH may serve as a model for cross-vendor implementation. S-PATH’s transparency and performance in external validation stand in contrast to the approach taken by many vendor-derived machine learning models, which often are proprietary and poorly validated.^44^

### Limitations

Nonetheless, this study also has some limitations. We observed some variation in S-PATH performance across hospitals, especially among hospitals with lower surgical volumes. Although the S-PATH AUROC was greater than 0.91 for more than 75% of the hospitals examined, there were a small number of hospitals with lower overall discrimination. In addition, the extent to which S-PATH outperformed the MSBOS approach varied across the hospitals. The reasons for this variation are unclear. Smaller hospitals may have different practice patterns than larger hospitals, including variability in case mix, less access to transfusion resources, and different transfusion preferences. However, we did not observe statistically significant correlations between hospital-level patient or case complexity or transfusion frequency and either of the primary or secondary outcomes. Taken together, these results suggest that model validation within local contexts will continue to be necessary before implementation of S-PATH as clinical decision support, especially for smaller hospitals.

Although this study was conducted within a national data registry, major academic medical centers were overrepresented in this sample, and the included community hospitals were largely from the state of Michigan, so our results may not generalize to other contexts. S-PATH does not include all the variables that potentially contribute to transfusion risk; for example, medications, genetic disorders, anatomical proximity to major vasculature, and surgeon skill or preference were not included. However, we believe the model provides a reasonable starting point for risk stratification that clinicians can modify given additional information. We examined the potential contribution of surgeon-level adjustment; use of surgeon-specific, procedure-specific historical transfusion rates instead of hospital-specific, procedure-specific transfusion rates did not meaningfully improve S-PATH performance (eTable 5 in Supplement 1). S-PATH was trained based on observed transfusion practice; therefore, any unconscious biases that clinicians may have had in their decisions to transfuse may have been learned by the model. However, we evaluated the possibility of racial bias and did not find meaningful differences (eFigure 3, eTable 3 in Supplement 1). Additionally, although it would have been ideal to compare S-PATH with observed type and screen ordering behavior, this information was not reliably available in MPOG. However, previous studies have indicated that more than 50% of surgical patients have type and screen orders,^23,45^ which is even higher than the baseline MSBOS model would suggest,^21^ so the difference between S-PATH and the baseline MSBOS model measured in this study likely underestimates the difference in type and screen orders compared with usual care.

## Conclusions

In this cohort study of 45 hospitals, a personalized surgical transfusion risk prediction algorithm demonstrated excellent external validity and discrimination. Despite the promise of machine learning in health care, only a handful of largely vendor-disseminated models have reached widespread implementation. The pathway for scaling machine learning models toward implementation remains challenging, and lack of external validation and demonstration of clinical value remain key barriers. Toward this end, we focused on an important clinical problem—presurgical blood ordering—with relevance to several specialties within medicine, including surgery, anesthesiology, transfusion medicine, and informatics.

## Notes

Supplement 1. **eFigure 1.** Participant flow diagram **eFigure 2.** Calibration (S-PATH vs MSBOS) **eFigure 3.** S-PATH calibration by race **eFigure 4.** Correlation between hospital characteristics and performance **eTable 1.** Distribution of S-PATH input variables **eTable 2.** Missingness of S-PATH input variables **eTable 3.** Performance by race **eTable 4.** Pearson correlations between hospital characteristics and performance **eTable 5.** Effect of surgeon-level adjustment

Supplement 2.
Nonauthor Collaborators

Supplement 3.
Data Sharing Statement
