# Supplementary material for: Multicenter Validation of a Machine Learning Model for Surgical Transfusion Risk at 45 US Hospitals
Source: JAMA Netw Open. 2025 Jun 27;8(6):e2517760. doi: 10.1001/jamanetworkopen.2025.17760 (PMC12205404; doi:10.1001/jamanetworkopen.2025.17760)
Supplement: Supplement 1. — eFigure 1. Participant flow diagram eFigure 2. Calibration (S-PATH vs MSBOS) eFigure 3. S-PATH calibration by race eFigure 4. Correlation between hospital characteristics and performance eTable 1. Distribution of S-PATH input variables eTable 2. Missingness of S-PATH input variables eTable 3. Performance by race eTable 4. Pearson correlations between hospital characteristics and performance eTable 5. Effect of surgeon-level adjustment [file jamanetwopen-e2517760-s001.pdf]

Supplemental Online Content

Lou SS, Kumar S, Goss CW, Avidan MS, Kheterpal S, Kannampallil T; Multicenter Perioperative Outcomes Group. Multicenter validation of a machine learning model for surgical transfusion risk at 45 US hospitals. *JAMA Netw Open*. 2025;8(6):e2517760. doi:10.1001/jamanetworkopen.2025.17760

eFigure 1. Participant flow diagram

eFigure 2. Calibration (S-PATH vs MSBOS)

eFigure 3. S-PATH calibration by race

eFigure 4. Correlation between hospital characteristics and performance

eTable 1. Distribution of S-PATH input variables

eTable 2. Missingness of S-PATH input variables

eTable 3. Performance by race

eTable 4. Pearson correlations between hospital characteristics and performance

eTable 5. Effect of surgeon-level adjustment

This supplemental material has been provided by the authors to give readers additional information about their work.

© 2025 Lou SS et al. *JAMA Network Open.*

eFigure 1 – Participant flow diagram illustrating inclusion and exclusion criteria for the validation cohort used for evaluating S-PATH performance.

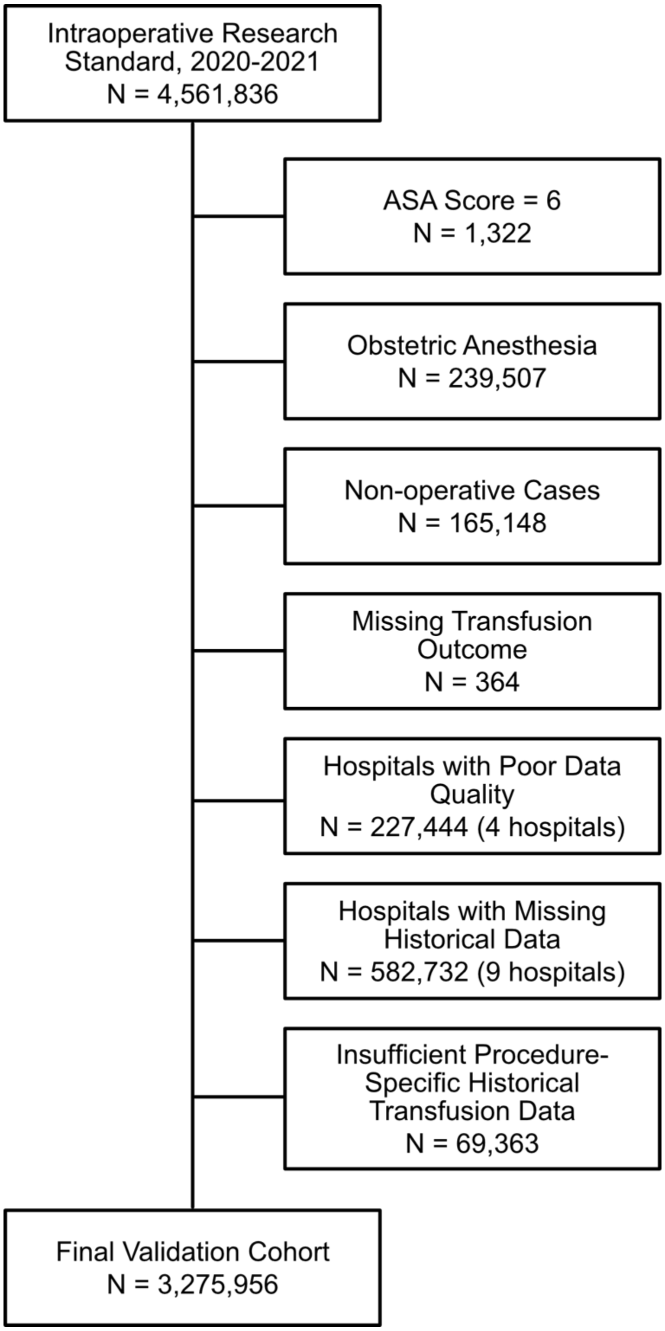

© 2025 Lou SS et al. *JAMA Network Open.*

eFigure 2 – Calibration plot illustrating the relationship between S-PATH predicted probabilities of transfusion and observed frequencies of transfusion compared with the standard of care MSBOS approach. Solid lines and dots show S-PATH (blue) and MSBOS (red) calibration across the entire validation cohort. Dashed black line indicates perfect calibration. The MSBOS approach, which use the historical hospital-specific procedure-specific transfusion rates as the predicted risk, is well-calibrated, indicating low drift in procedure-specific transfusion rates over time. The S-PATH approach has a mild tendency to overestimate risk, which is likely safer than underestimating risk.

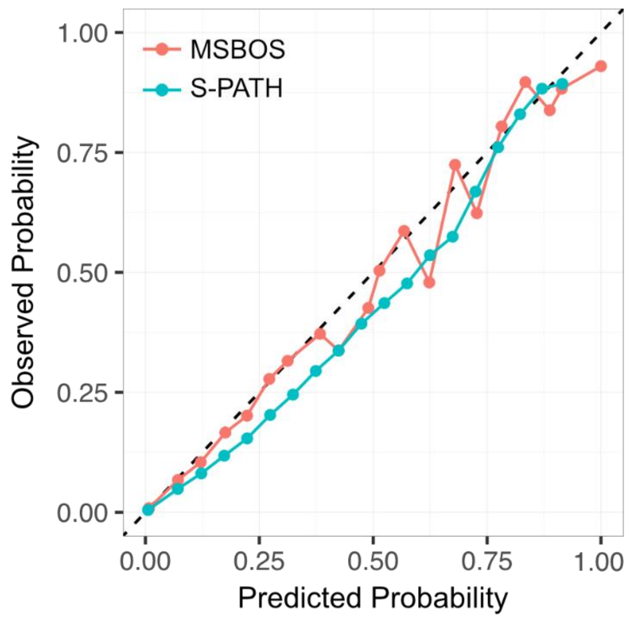

eFigure 3 – S-PATH calibration stratified by race across the entire validation cohort (N = 3,275,956). Dashed black line indicates perfect calibration.

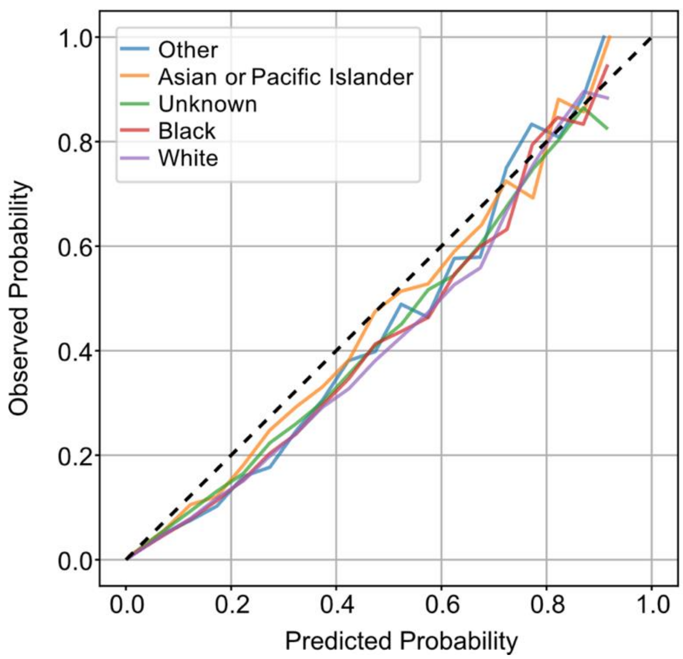

© 2025 Lou SS et al. *JAMA Network Open.*

eFigure 4 – Correlation between hospital-level characteristics and S-PATH performance. Each hospital is shown as an individual dot. Hospital-level characteristics are shown on the x-axis and S-PATH performance metrics are shown on the y-axis. P-values are only shown on the plot if they were statistically significant. Otherwise, the list of correlation coefficients and p-values are provided in Supplemental Table 4. ASA Score refers to ASA Physical Status classification, a measure of patient complexity. Base Units are a measure of procedural complexity. Transfusion Quality refers to adherence to MPOG transfusion quality metric TRAN01, which measures the frequency of checking a hemoglobin or hematocrit within 90 minutes of transfusion.

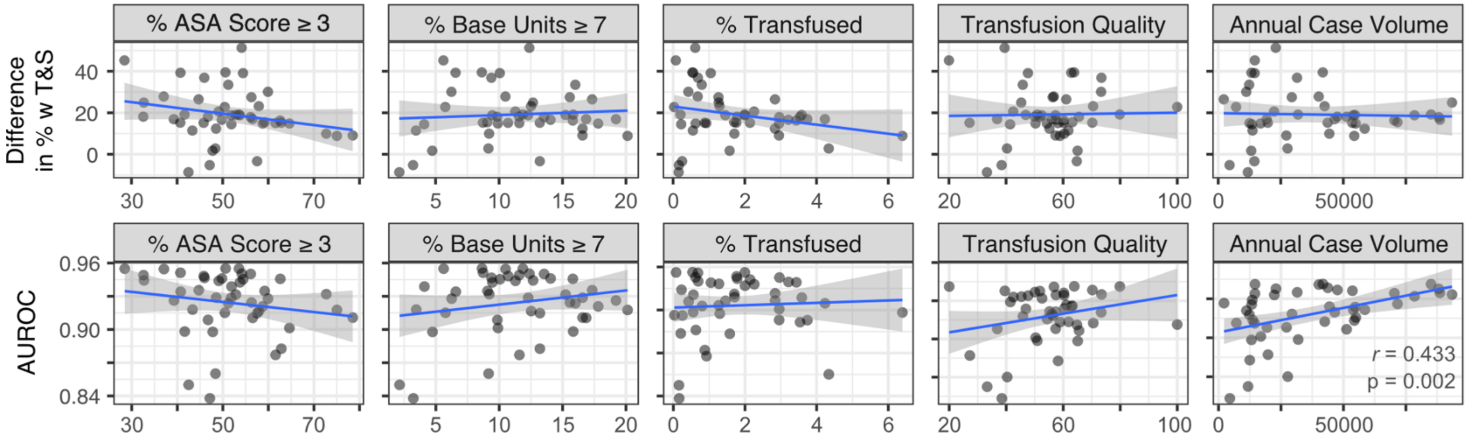

© 2025 Lou SS et al. *JAMA Network Open.*

eTable 1 – Distribution of the input variables for S-PATH at the 45 included hospitals in the validation cohort. For continuous variables, the median value was calculated at each hospital, and the median and interquartile range of this median is shown. For categorical variables, the percentage in each category was calculated at each hospital and the median and interquartile range of this percentage is shown.

Variable	Data Type	Median (IQR)
**Age** (years)	Continuous	59 (57 – 60)
**Height** (in)	Continuous	67 (67 – 67)
**Weight** (lbs)	Continuous	177 (170 – 184)
**Sex** – Male (%)	Binary	46 (44 – 48)
**Hypertension** – Yes (%)	Binary	11 (9 – 14)
**Congestive heart failure** – Yes (%)	Binary	8 (6 – 10)
**Smoking** – Yes (%)	Binary	6 (3 – 12)
**COPD** – Yes (%)	Binary	13 (10 – 19)
**Dialysis** – Yes (%)	Binary	1.5 (1.2 – 2.1)
**Diabetes** – Yes (%)	Binary	5 (4 – 6)
**Hematocrit**, g/dL	Continuous	39 (38 – 40)
**Platelet**, 1000/uL	Continuous	243 (239 – 247)
**INR**	Continuous	1.1 (1.1 – 1.1)
**PTT**, s	Continuous	31 (30 – 32)
**Creatinine**, mg/dL	Continuous	0.87 (0.85 – 0.88)
**Sodium**, mEq/L	Continuous	139 (138 – 140)
**Albumin**, g/dL	Continuous	4.1 (4.0 – 4.2)
**Bilirubin**, mg/dL	Continuous	0.5 (0.5 – 0.6)
**Elective surgery** – Yes (%)	Binary	92 (89 – 94)
**Procedure-specific transfusion rate**, %	Continuous	0.29 (0.15 – 0.45)

© 2025 Lou SS et al. *JAMA Network Open.*

eTable 2 – Rates of missingness for the input variables for S-PATH for hospitals in the validation cohort. The percentage of missingness was calculated at each hospital, and the median and IQR for the percentage missingness across all the hospitals is shown. Missing values were subsequently imputed using the S-PATH pipeline as described in the methods section (using median values from the original NSQIP cohort used for model training).

Variable	Missingness % (median, IQR)
**Age**	0 (0 – 0)
**Height**	0.8 (0.4 – 2.4)
**Weight**	0.6 (0.3 – 1.4)
**Sex**	0 (0 – 0)
**Hypertension**	0.1 (0.1 – 4.5)
**Congestive heart failure**	0.1 (0.1 – 4.5)
**Smoking**	0 (0 – 0)
**COPD**	0.1 (0.1 – 4.5)
**Dialysis**	0.1 (0.1 – 4.5)
**Diabetes**	0.1 (0.1 – 4.5)
**Hematocrit**	30 (26 – 36)
**Platelet**	31 (27 – 40)
**INR**	62 (60 – 71)
**PTT**	79 (70 – 86)
**Creatinine**	31 (25 – 35)
**Sodium**	31 (25 – 37)
**Albumin**	46 (39 – 56)
**Bilirubin**	44 (39 – 54)
**Elective surgery**	0 (0 – 0)
**Procedure-specific transfusion rate**	0 (0 – 0)

© 2025 Lou SS et al. *JAMA Network Open.*

eTable 3 – S-PATH performance stratified by race at each individual hospital. Median (IQR) is shown for all metrics across the 45 included hospitals.

Race	N hosp	N patients	Median Hct (%)	% Transfused	AUROC	Sensitivity	% w T&S
**White**	44	2,342,837 (71%)	39.7 39.1-40.0	0.013 0.006-0.027	0.930 (0.915-0.952)	0.960 (0.955-0.964)	0.316 (0.252-0.420)
**Black**	44	404,191 (12%)	37.0 36.5-37.7	0.020 0.008-0.036	0.919 (0.884-0.929)	0.953 (0.945-0.971)	0.366 (0.318-0.454)
**Unknown**	45	302,850 (9%)	38.9 38.0-39.8	0.015 0.005-0.029	0.931 (0.904-0.946)	0.954 (0.934-0.971)	0.313 (0.248-0.383)
**Asian Pacific**	44	124,647 (4%)	38.8 38.3-39.6	0.018 0.006-0.028	0.935 (0.922-0.960)	0.956 (0.908-1.000)	0.306 (0.233-0.363)
**Other**	43	101,431 (3%)	38.9 38.0-39.8	0.014 0.003-0.028	0.942 (0.910-0.963)	0.974 (0.951-1.000)	0.310 (0.267-0.434)

eTable 4 – Pearson correlation coefficients and associated p-values for the relationship between the indicated hospital-level characteristics and S-PATH performance metrics. * indicates p < 0.05.

**Hospital-level characteristic**	**Pearson's *r*, Difference in % with T&S**	**P-value**	**Pearson's *r*, AUROC**	**P-value**
Annual Case Volume	-0.034	0.825	0.456	0.002 *
% Cases Transfused	-0.249	0.098	0.056	0.714
% ASA Score ≥3	-0.234	0.122	-0.171	0.261
% Base Unit ≥7	0.076	0.622	0.204	0.178
% Passing TRAN01	0.022	0.886	0.246	0.103

© 2025 Lou SS et al. *JAMA Network Open.*

eTable 5 – Effect of using surgeon-specific procedure-specific historical transfusion rates versus hospital-specific procedure-specific historical transfusion rates on S-PATH performance. Surgeon-specific procedure-specific transfusion rates were calculated using the historical cohort. Only surgeon-procedure combinations in the validation cohort with at least 50 historical examples were included in this analysis (N = 43 institutions and 1,431,806 cases). S-PATH performance was evaluated in this limited cohort using the surgeon-specific procedure-specific transfusion rates and again using the hospital-specific surgeon-specific transfusion rates. Aggregate performance across the entire cohort is shown, along with 95% confidence intervals generated by bootstrap resampling.

	**AUROC**	**Sensitivity**	**% w T/S**
Hospital-level priors	0.943 (0.939, 0.947)	0.959 (0.950, 0.967)	0.274 (0.271, 0.277)
Surgeon-level priors	0.946 (0.942, 0.950)	0.960 (0.952, 0.968)	0.284 (0.281, 0.287)

© 2025 Lou SS et al. *JAMA Network Open.*
