# Supplementary material for: Multicenter Validation of a Machine Learning Model for Surgical Transfusion Risk at 45 US Hospitals
Source: JAMA Netw Open. 2025 Jun 27;8(6):e2517760. doi: 10.1001/jamanetworkopen.2025.17760 (PMC12205404; doi:10.1001/jamanetworkopen.2025.17760)
Supplement: Supplement 2. — Nonauthor Collaborators [file jamanetwopen-e2517760-s002.pdf]

Supplemental Online Content: Nonauthor Collaborators
*First name, last name, and suffix (if applicable) are required and will appear in PubMed.

*Group Name(s): Multicenter Perioperative Outcomes Group							
***First Name and Middle Initial(s)**	***Last Name**	***Suffix (eg, Jr, III)**	Academic Degrees	Institution	Location (city, state/province, country)	Role or Contribution, eg, chair, principal investigator	Group (if more than 1 Group listed in the byline) and/or Subgroup (eg, Steering Committee)
Robert E	Freundlich		MD, MS	Vanderbilt University	Nashville, TN	Non-author collaborator	
Michael R	Mathis		MD	University of Michigan	Ann Arbor, MI	Non-author collaborator	
Vikas N	O'Reilly-Shah		MD, PhD	University of Washington	Seattle, WA	Non-author collaborator	
Kunal	Karamchandani		MD, FCCP,	University of Texas Southwestern	Dallas, TX	Non-author collaborator	
