# Supplementary material for: Multicenter Validation of a Machine Learning Model for Surgical Transfusion Risk at 45 US Hospitals
Source: JAMA Netw Open. 2025 Jun 27;8(6):e2517760. doi: 10.1001/jamanetworkopen.2025.17760 (PMC12205404; doi:10.1001/jamanetworkopen.2025.17760)
Supplement: Supplement 3. — Data Sharing Statement [file jamanetwopen-e2517760-s003.pdf]

Data Sharing Statement

Lou. Multicenter Validation of a Machine Learning Model for Surgical Transfusion Risk at 45 US Hospitals. *JAMA Netw Open*. Published June 27, 2025.
doi:10.1001/jamanetworkopen.2025.17760

Data

Data available: The datasets involved in this study are defined as limited datasets per United States Federal Regulations and require execution of a data use agreement for transfer or use of the data. They are derived from data shared within the Multicenter Perioperative Outcomes Group (MPOG). The investigative team is able to share data securely and transparently conditional on: (i) receipt of a detailed written request identifying the requestor, purpose and proposed use of the shared data, (ii) use of a secure enclave for the sharing of personally identifiable information and (iii) the request is permissible within the confines of existing data use agreements executed between MPOG members.
